# Welcome to volume 2 of *Future Science OA*

**DOI:** 10.4155/fso.15.98

**Published:** 2016-02-03

**Authors:** Francesca Lake

**Affiliations:** 1 Future Medicine Ltd, Unitec House, London, N3 1QB, UK

**Figure 1.**
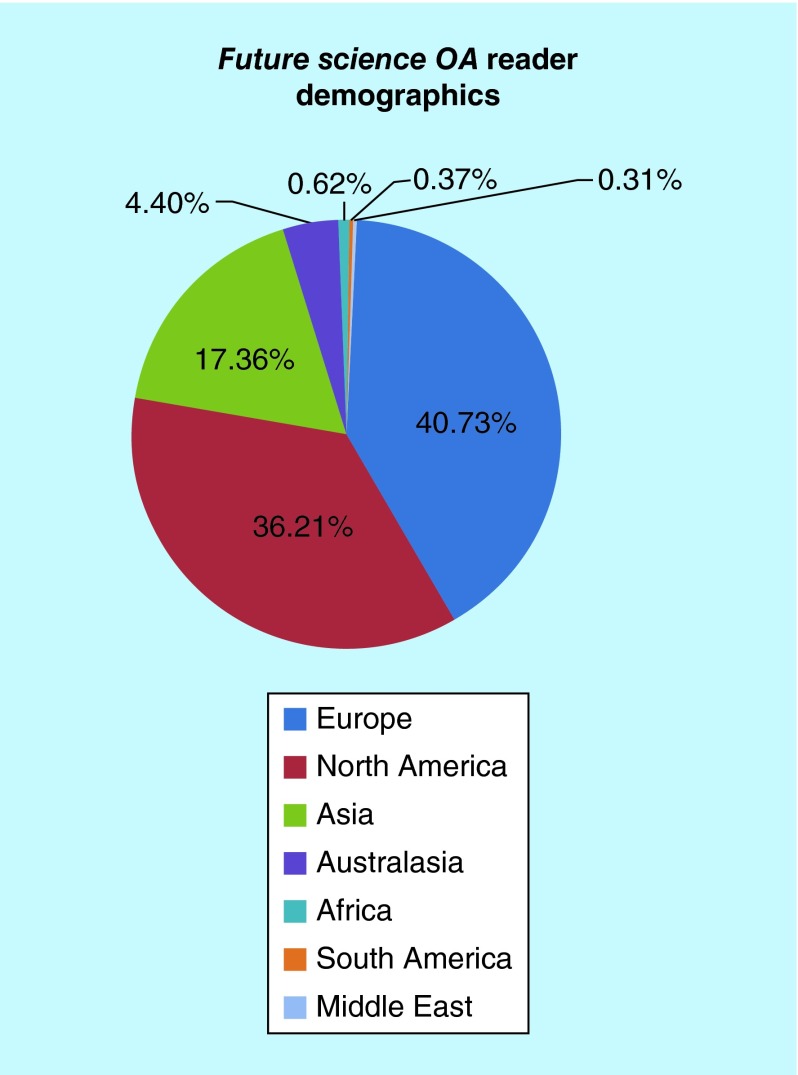
***Future Science OA* reader demographics.**

**Figure 2.**
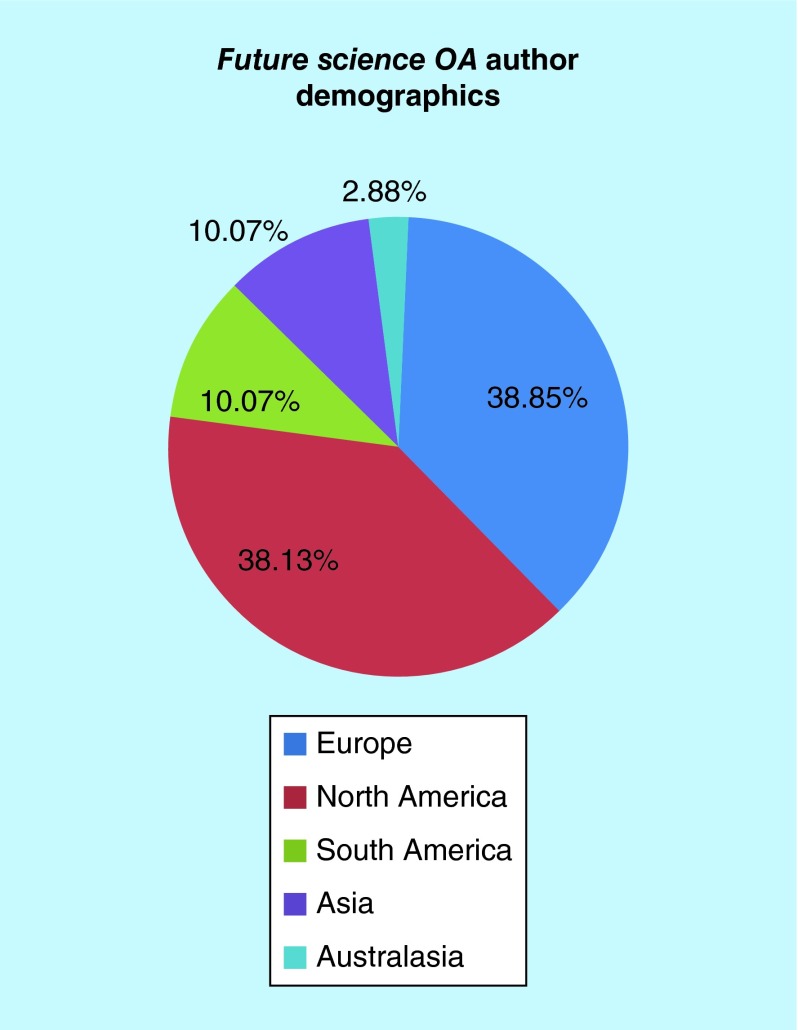
***Future Science OA* author demographics.**

Welcome to volume 2 of *Future Science OA*. 2015 was an exciting year for us, with the publication of our first content and being indexed by the Database of Open Access Journals [1], and we discuss our highlights from the past year in this Foreword. We would like to take this opportunity to thank our editorial board, authors, peer reviewers and readers for their contributions thus far. We look forward to working with you all over the coming year.

## Content highlights from 2015

We published over 70 articles in 2015, which comprised editorials, opinion pieces, reviews and original research from an international group of authors. Covering a wide range of topics of relevance to medicine, we have seen over 2500 full-text readers each month, from across the globe. The majority of our readers are within North America, Europe and Asia, with readers also across Australasia, South America, the Middle East and Africa (Figure 1). This is a pattern also seen in our authorship, with two-thirds of our authors residing in Europe and North America (Figure 2). In 2016, we are excited to be partnering with Enago [2], who will provide our authors for whom English is a second language with pre-submission editing and translation services. We hope this will further expand our publications from countries around the globe.

Our most-read article saw Françoise Barré-Sinoussi and Xavier Montagutelli (Institut Pasteur, France) provide an insight into their thoughts on the future of animal models in biological research [3], following the ‘Stop Vivisection’ European Citizen Initiative presented to the EC. Covering many of the issues facing the use (and potential future non-use) of animals in research, they presented why they feel animal use will remain an essential feature of research, in the near future at least.

With the increasing prevalence of Alzheimer's disease and the continued lack of an effective therapy, Ruth MacLeod and colleagues (University of Glasgow, UK) reviewed the role and therapeutic targeting of secretases in Alzheimer's disease [4], asking whether targeting amyloid is a viable hypothesis.

Mariana Varna and colleagues (Inserm-X Bichat Hospital, France) reviewed current work looking into the potential of nanomedicine to help solve the issue of inactivation of thrombolytic drugs in the blood [5]. They eagerly await validation of nanosystems in this arena ready for the clinic. In further nanomedicine news, Jun Fang and colleagues (Sojo University, Japan) presented research examining the potential of a tumor-targeted nanoprobe for photodynamic therapy and tumor imaging [6].

Gaurav Agrawal, Thomas Borody and colleagues (Centre for Digestive Diseases, Australia) presented a case report evaluating a novel combination therapy for resistant fistulizing Crohn's disease [7].

Other top articles to highlight include Andreea Milasan and colleagues’ review of the lymphatic network in atherosclerosis [8], Bejoy Thomas *et al*.'s case report demonstrating a need for more collaboration to ensure literature reviews are comprehensive [9], a new generic model to help the development of applications for clinical decision-making created by Tibor van Rooij and colleagues [10], an examination of the potential uses of the STAT3 pathway in genitourinary tumors [11], and, finally, a research article examining the potential of extracellular embryo genomic DNA for genotyping applications [12]. This content is merely a snapshot of our excellent articles, and once again we thank all of our authors for their efforts throughout the year.

## Special issues

We published two excellent special issues in 2015. The first examined detection and delivery of nitric oxide releasing materials, featuring guest editors Joel and Adam Friedman (Albert Einstein College of Medicine, USA) [13]. The second, featuring guest editor Salvador Ventura (Universitat Autònoma de Barcelona, Spain), provided a snapshot into protein misfolding diseases, aiming to collate information on underlying causes and provide insight into potential future therapeutic strategies [14]. We look forward to publishing further issues, and welcome topic suggestions from our readers.

## Editorial board

We have been very thankful to the editorial board [15] for their help in creating *Future Science OA*, and their contributions to the journal thus far. Our senior editors Benoit Arsenault (Université Laval, Canada), Ian A Blair (University of Pennsylvania, PA, USA) and Paul Span (Radboud University Nijmegen Medical Centre, The Netherlands) are joined by international experts from across the globe. In addition, we are supported by a panel of young ambassadors; early-career researchers who are helping us to develop the journal to ensure it meets the needs of the next generation of researchers.

## Social media

For those of you active on social media, you may be aware of our relevant accounts, through which we keep you abreast of journal content and news, and encourage discussion of our articles. If you have not already, you can follow us on LinkedIn [16] and Facebook [17], and on Twitter at @fsgfso.

## Improving article discoverability

The open access nature of *Future Science OA* allows us to ensure we maximize discoverability and readership of our articles. All of our articles are released through a variety of online outlets, and this year we have implemented a variety of ways to aid our authors in increasing the reach of their work.

We have been delighted to begin working with Altmetric [18], which allows our authors and readers to see the online discussion surrounding our articles. Each article page now features an Altmetric badge to this effect, where you can see where our articles are being discussed.

In addition, we have partnered with Kudos [19] to aid our authors in improving the discoverability of their own articles, and to further track their article success. You can find a full guide covering how to improve discoverability of your article on our webpage [20].

## Conclusion

2015 was an excellent year, and we look forward to working with you all in 2016. We welcome unsolicited article proposals covering all topics of relevance to human health, and would be delighted to hear from you. Finally, if you have any suggestions about the journal, please do not hesitate to get in touch.
